# Gum Arabic (*Acacia Senegal*) Augmented Total Antioxidant Capacity and Reduced C-Reactive Protein among Haemodialysis Patients in Phase II Trial

**DOI:** 10.1155/2020/7214673

**Published:** 2020-04-09

**Authors:** Nour Elkhair Ali, Lamis AbdelGadir Kaddam, Suad Yousif Alkarib, Babikir Gabir Kaballo, Sami Ahmed Khalid, Abdalazim Higawee, Alaa AbdElhabib, Alaa AlaaAldeen, Aled O. Phillips, Amal Mahmoud Saeed

**Affiliations:** ^1^Department of Physiology, Faculty of Medicine, Alneelain University, Khartoum, Sudan; ^2^Nephrology Unit, Military Hospital Omdurman, Sudan; ^3^Department of Pharmaceutics, College of Pharmacy, Karary University, Khartoum, Sudan; ^4^Department of Medicine, Medicine & Surgery College, Karary University, Khartoum, Sudan; ^5^Faculty of Pharmacy, University of Science & Technology, Omdurman, Sudan; ^6^Institute of Nephrology, Cardiff University School of Medicine, Cardiff, UK; ^7^Department of Physiology, Faculty of Medicine, University of Khartoum, Khartoum, Sudan

## Abstract

**Background:**

Oxidative processes might increase in patients with end-stage renal disease (ESRD) according to the current literature. Oxidative stress (OS) is a risk factor of atherosclerosis and cardiovascular complications, which are major causes of mortality among ESRD patients. Haemodialysis (HD) is life-saving procedure, nevertheless it is an active chronic inflammatory status that could augment cardiovascular disease and increase mortality. Gum Arabic (GA) has been claimed to act as an antioxidant and anti-inflammatory agent in experimental studies and clinical trials. Therefore, we assumed GA supplementation among haemodialysis patients would reduce oxidative stress and consequently reduce the state of chronic inflammatory activation associated with haemodialysis.

**Methods:**

Forty end-stage renal failure (ESRF) patients aged 18–80 years who were on regular haemodialysis in Arif Renal Center, Omdurman, Sudan, were recruited. All recruited patients met the inclusion criteria and signed informed consent prior to enrolment. The patients received 30 g/day of GA for 12 weeks. C-reactive protein (CRP) and complete blood count (CBC) were measured as baseline and monthly. Total antioxidant capacity (TAC) and oxidative stress marker malondialdehyde (MDA) levels were measured before and after GA intake. Ethical approval from the National Medicines and Poisons Board was obtained.

**Results:**

Gum Arabic significantly augmented total antioxidant capacity level (*P* < 0.001) (95% CI, 0.408–0.625) and also attenuated oxidative marker MDA and C-reactive protein (*P* < 0.001).

**Conclusions:**

GA has revealed potent antioxidative and anti-inflammatory properties in haemodialysis patients. Oral digestion of GA (30 g/day) decreased oxidative stress and inflammatory markers among haemodialysis patients. Trial registration. ClinicalTrials.gov Identifier: NCT03214692, registered 11 July 2017 (prospective registration).

## 1. Introduction

Oxidative stress results from disparity between systemic manifestation of reactive oxygen species and a body ability either to eradicate reactive intermediates or to repair cellular damage [1]. Due to metabolic process, cells constantly produce free radicals and reactive oxygen species (ROS). The latter are counteracted by the antioxidant defence system composed of enzymatic and nonenzymatic antioxidants, e.g., vitamins A, E, and C and glutathione [2]. Oxidative stress (OS) has been connected to various diseases' pathogenesis, such as renal disease, cardiovascular disease, atherosclerosis, hypertension, cancer, diabetes, and aging [3, 4].

OS increased in haemodialysis patients due to deficiency of dietary exogenous antioxidants, accumulation of oxidative products, and drain of antioxidant elements during the dialysis process [5]. All these factors eventually results in the development of chronic inflammation, atherosclerosis, and cardiovascular diseases (CVD) [5]. CVD is the leading cause of mortality among ESRD patients who receive renal replacement therapy [6, 7]. Hence, these events are responsible cause of death for 34% of HD patients [7]. Other contributing factors are increased lipid peroxidation and antioxidants exhaustion which increase the risk of atherosclerosis [6, 7].

OS has a major role in renal damage; therefore, it is a potential focus for therapeutic strategies [8]. Several attempts are considered to apply oral and/or intravenous antioxidants to attenuate or prevent the inflammatory status, CVD, and the subsequent impact of these events on the mortality. Alpha-tocopherol, vitamin C, and *N*-acetylcysteine are considered among the most commonly used antioxidants to counteract OS [8]. Among them, *N*-acetylcysteine seems to be the most efficacious agent [5]. Recently, curcumin was found to attenuate OS among experimental rats with diabetic nephropathy [9].

Gum Arabic (GA) is an *Acacia senegal* exudate as defined by the FAO/WHO Joint Expert Committee for Food Additives (JECFA) [10]. GA cannot be digested either by humans or by animals [11]. It is a heteropolysaccharide [12, 13] and contains minerals like calcium, potassium, and magnesium [14]. In addition, GA has less than 3% protein and fewer amount of nitrogen [15]. Serine, hydroxyproline, proline, and aspartic acid are the major amino acids present in GA [15]. It is highly water soluble [13] but insoluble in alcohol [16].

Gum Arabic (GA) supplementation has been implicated with its beneficial effect on CKD patients for quite a long time [17]. GA has been used as part of Sudanese traditional medicine to treat chronic renal failure (CRF) long time ago [15]. It seems to have the potential to modify the human physiological status beneficially [15]. Few studies have revealed its positive effect on inflammatory status and oxidative stress in animal models [18] as well as clinical studies involving CKD patients quite recently [19]. GA oral ingestion was associated with a significant reduction in C-reactive protein (CRP) level among CKD patients [19].

In fact, GA considered as prebiotic fibres since oral intake increases serum short-chain fatty acid serum level [14, 20]. GA has good reputation and promising effects as anticancer [21], antimalarial [22], immune-modulatory [23], and antioxidant [18, 24–26] agents.

The protective role of GA against nephrotoxicity includes significant increase in creatinine clearance, suggesting favourable actions in renal insufficiency [27, 28]. The suggested mechanism of action, based on animal studies, is as follows: GA increases nitrogen excretion in faeces and decreases serum urea concentration among CRF patients [17, 28–31], subsequently reducing dialysis sessions twice per week instead of three with dramatic positive economic and psychological impact [32]. Previous study from Central Sudan revealed 50 g/day of GA supplementation improved biochemical profile of haemodialysis patients [17, 32].

GA decreased MDA and superoxide production significantly and increased GSH and TAC levels among rats with induced nephrotoxicity and CRF [24, 33]. GA antioxidant capacity could be attributed to its amino acids [34]. In 2017, Kaddam et al. reported a novel effect of GA as an antioxidative agent among humans since it increases TAC level and decreases oxidative stress markers among sickle cell anaemic patients [26].

GA beneficial effects may also be intermediated by its immune-modulatory and anti-inflammatory actions [23, 35, 36]. GA modulates immunity in mice [23] through attenuating TNFα and CRP and increasing anti-inflammatory cytokine IL10 [23, 27, 37]. GA exerted local anti-inflammatory effects by modifying nuclear factor-*κ*B (NF-*κ*B) on the small intestine [38].

Generalized increase in oxidative stress associated with uraemia has led to the suggestion that antioxidative therapy may have a role in clinical setting [39]. Dietary soluble fibre supplementation can attenuate inflammatory and oxidant status among haemodialysis patients. Dietary fibre supplementation could be one of significant preventative strategies to decrease CVD mortality and morbidity among haemodialysis patients.

To the best of our knowledge, this is the first attempt to address utilization of GA as an antioxidant and anti-inflammatory agent among haemodialysis patients.

## 2. Material and Methods

### 2.1. Study Subjects

A single-arm nonrandomized open-label clinical trial was conducted among (ESRD) adult patients (their GFR was less than 15 ml/min/1.73 m^2^) on regular HD in Alshaheed Arif Renal Disease Centre in Military Hospital, Khartoum, Sudan. The inclusion criteria were end-stage renal disease patients on regular haemodialysis over three months and aged above 18 years old. The exclusion criteria were as follows: patient known to have ischemic heart disease, pregnant women, patients who had impaired liver function or hepatitis virus or/and HIV positive, and patients currently using Gum Arabic or/and lipid lowering-agents.

### 2.2. Gum Arabic Administration

GA was provided in powder form by Gum Arabic Company, Khartoum, Sudan. The GA powder was a pure extract produced mechanically from the wildly grown *Acacia senegal* tree with no additives. Daily dose was 30 gram. The dose was chosen based on earlier studies [26]. GA was packed as 15 g/sachet. Two sachets were to be dissolved in water and consumed early morning on empty stomach. Participants received 56 sachets monthly for twelve weeks. To validate compliance, empty sachets were retained.

### 2.3. Measurements

#### 2.3.1. Sample Collection and Analysis

Blood samples were collected before administering GA after 4, 8, and 12 weeks as follows: 2 ml in an EDTA container and 3 ml in a plain container. Serum was separated by centrifugation at 3000 rpm for 15 minutes and stored at −85°C. A certified nurse withdrew all blood samples immediately before the dialysis session.

To determine the TAC of the samples, 0.5 ml of H_2_O_2_ was added to 20 *μ*l of serum and incubated for 5 min at 37°C. The principal of test depends on the reaction of total antioxidants with exogenous hydrogen peroxide (H_2_O_2_). The remaining H_2_O_2_ was determined calorimetrically by an enzymatic reaction which included the conversion of 3,5-dichloro-2-hydroxybenzenesulfonate to a colored product [40].

MDA level was measured by using thiobarbituric acid reactive species (TBARS) technique [41]. 200 *μ*l of serum was mixed well in the test tube with 1 ml of TBA and boiled in boiling water bath (95°C) for half an hour. The absorbance of sample against blank and standard against distilled water was read at 534 nm [41].

Quantitative estimation of CRP level was done using MISPA i_2_ (AGAPPE Diagnostics GmbH, Switzerland) following nephelometry [42]. The reagent composition is CRP R1 (glycine buffer) and CRP R2 latex suspension coated with anti-CRP antibodies (rabbit polyclonal antibody) with the lower detection limit of 0.5 mg/l (highly sensitive assay). TAC and MDA were measured before and after completion of the trial, while CRP and blood count were measured on monthly basis.

### 2.4. Statistical Analysis

Data were analyzed using univariate and bivariate analysis using GraphPad prism version 7. Quantitative data were expressed as mean and standard deviation. Repeated measures analysis of variance (ANOVA) was used to analyze the data recorded monthly. Data were adjusted with Bonferroni correction. We choose paired *T* test to compare pre- and postintervention results. *P* value equal to or less than 0.05 was considered significant.

## 3. Results

### 3.1. Patient Enrolment and Study Duration

**Figure 1:**
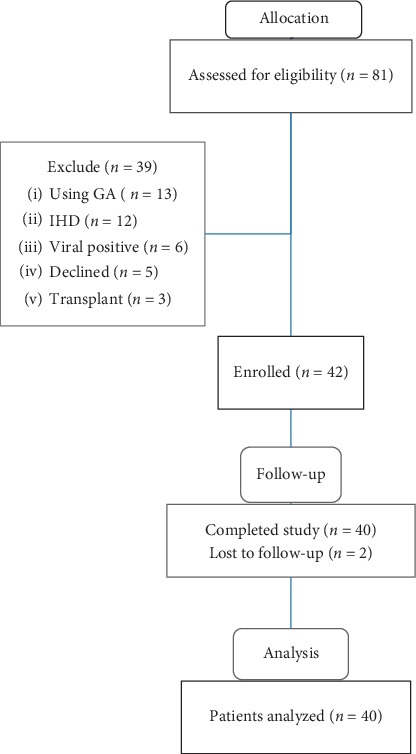
Patients' recruitment and allocation in the clinical trial.

**Figure 2:**
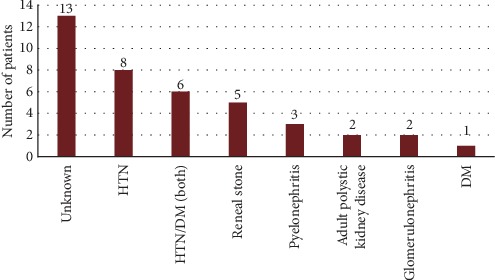
Causes of ESRD among study population. HTN, hypertension; DM, diabetes mellitus.

We screened all patients who were under regular haemodialysis at Alshaheed Arif Renal Disease Centre, Military Hospital in Khartoum, started on July 2017 aiming for total coverage. We identified 81 patients with ESRD and excluded 39 patients for various reasons (Figure 1). All were Sudanese; 24 were males and 16 females, and their age ranged from 20 to 75 years. The cause of ESRD was unknown for one-third of participants followed by hypertension (Figure 2).

Duration of treatment was twelve weeks except one patient who received GA for ten weeks.

Forty patients presented for follow-up and were included in the final analysis; their baseline characteristics are outlined in Table 1.

**Figure 3:**
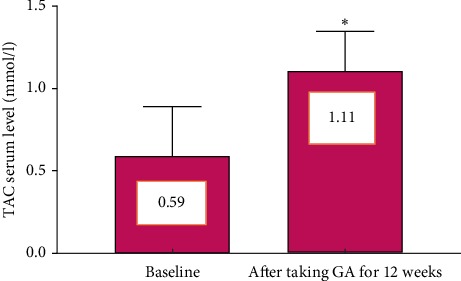
Effect of GA intake on TAC level (*P* < 0.001). Bars represent mean ± SD. ^*∗*^Significant difference from baseline.

**Figure 4:**
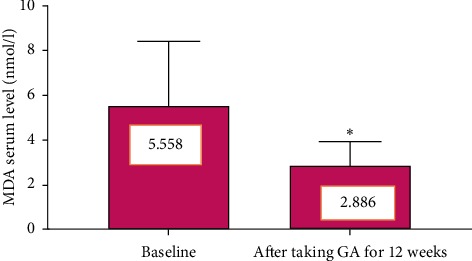
Effect of GA intake on MDA level (*P* < 0.001). Bars represent mean ± SD. ^*∗*^Significant difference from baseline.

**Figure 5:**
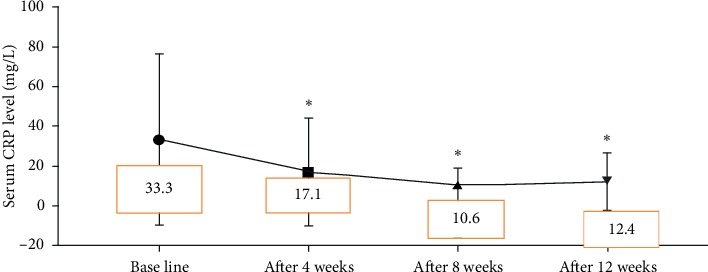
Effect of GA intake on CRP level (*P* < 0.001). ^*∗*^Significant difference from baseline.

Oral Gum Arabic intake significantly augmented the level of TAC (Figure 3) and reduced the levels of both MDA (Figure 4) and CRP (Figure 5). GA significantly increased haemoglobin level and RBC count (Table 2).

### 3.2. GA Tolerance and Side Effects

More than 50% of patients reported gastrointestinal symptoms such as bloating, diarrhoea, nausea, and vomiting. All these symptoms resolved spontaneously within the first two weeks. Compliance to GA was excellent, evidenced by follow-up and the retained number of empty sachets.

## 4. Discussion

Renal replacement therapy (haemodialysis) in end-stage renal disease (ESRD) is associated with amplified oxidative stress which involves in the pathogenesis of kidney failure events, systemic complications, and subsequently cardiovascular diseases [6, 8]. MDA and free radicals production increased in inflammatory kidney diseases including ESRD [4, 5]. Based on recent researches, OS may be mediated by myeloperoxidase (MPO), and the latter may have a role in development of cardiovascular complications in dialysis patients [6]. Moreover, dialysis treatment *per se* exaggerates OS [5]. Therefore, this study was designed to determine the effect of GA on malondialdehyde (MDA), which is considered as a marker of oxidative stress among ESRD patients on regular HD. Biomarkers of oxidative stress are altered in uremic patients [39]. These alterations include concentration of lipid peroxidation products, mainly MDA, which was found to be higher in uremic patients compared to healthy people [39, 43].

Results of this interventional clinical trial provided first clinical evidence to consolidate the effect of GA on oxidative stress and inflammatory status among haemodialysis patients. The results revealed that Gum Arabic supplementation for 12 weeks increased serum concentration of total antioxidant capacity (TAC) (Figure 3). Postsupplementation values of blood MDA content, which is a marker for oxidant substances released by dialysis, were significantly lowered compared to baseline values (*P* < 0.001) (Figure 4). GA significantly decreased MDA level in the CRF animal model and nephrotoxicity [18, 24]. An experimental study showed that Gum Arabic significantly reduced OS in sickle cell anemia patients [26].

In addition, we evaluated Gum Arabic's effect on inflammatory status in haemodialysis patients and found that CRP was significantly reduced on monthly basis (Figure 5). Haemodialysis is considered as an inflammatory state [44, 45] and causes of inflammation are multifactorial including blood exposure to dialyzer membranes or tubing, vascular access's infections, reduced antioxidants, and increased oxidative stress [46]. Alamin et al.'s study revealed that 10–40 gram/day of GA significantly reduced CRP level among CKD patients [19]. Recent research reported comparable results to our finding and stated that dietary soluble fibre can counteract inflammatory and malnutrition status among haemodialysis patients [47]. An early study showed GA significantly decreased TNFα level and ESR among rheumatoid patients [36].

There was significant increase in both haemoglobin concentration and RBC count in our study population (Table 2), which may attribute partially to regular administration of erythropoietin injection during the last 4 weeks. On the other hand, GA may increase the response to erythropoietin injection due to diminished CRP level. Inflammation is one of the major causes of resistance to erythropoietin (EPO) treatment [48]. No significant change on other blood indices, MCV and MCH, was detected. By contrast, a previous study indicated that GA intake increases MCV [49]. There was no significant difference in platelet count and TWBC. Similar results were recorded among sickle cell anemia and rheumatoid patients where GA revealed no effect on reticulocytes, RBCs, and platelets with insignificant decrease in WBCs [36, 49]. An early study conducted among healthy volunteers found that there is no influence of GA in blood indices [13].

Our results revealed promising outcome of GA supplementation among haemodialysis patients, manifested by significant decrease in oxidative stress and inflammatory markers, both considered as risk factors for atherosclerosis and cardiovascular diseases. In resource-limited settings, where patients have limited access to renal transplantation and relay mainly on dialysis for years to keep alive (Table 1), causes of ERSD were not recognized (Figure 2). GA could be utilized safely to ameliorate oxidative stress and inflammation induced by haemodialysis and renal disease itself. Nevertheless, testing the effect of GA on morbidity and mortality and development of atherosclerosis and ischemic heart diseases, longer and multiarm trials should be conducted to overcome current limitations such as small sample size and short period of intervention.

## 5. Conclusions

The present study revealed that GA intake significantly elevated TAC and inversely decreased MDA levels coupled with significant and concurrent decrease in CRP. In addition, GA increased haemoglobin level and RBC count significantly without substantial effect on other blood parameters.

## Figures and Tables

**Table 1:** Baseline characteristics of the study population (*n* = 40).

Parameter	Mean ± SD
Age (years)	45.5 ± 17.22
Sex (M/F)	26/14
Dialysis duration (months)	59.7 ± 38.38
Wight (kg)	57.94 ± 14.34

**Table 2:** Effect of Gum Arabic on blood parameters.

Parameter	Baseline level, mean ± SD	After 4 weeks, mean ± SD	After 8 weeks, mean ± SD	After12 weeks, mean ± SD	*P* value, mean ± SD
Hb	8.8 ± 2	8.2 ± 1.9	8.785 ± 1.69	9.541 ± 1.97	0.0357^*∗*^
MCV	85.85 ± 5.35	83.34 ± 13.58	85.55 ± 5.369	86.05 ± 5.27	0.4405
MCHC	31.89 ± 1.24	32 ± 1.4	31.6 ± 1.2	31.4 ± 1.2	0.1598
MCH	27.39 ± 2.12	27.34 ± 2.249	27.05 ± 2.2	27.05 ± 2.11	0.8492
WBCs	5.256 ± 1.962	5.497 ± 2.266	5.241 ± 2.09	5.869 ± 2.77	0.5916
RBCs	3.206 ± 0.83	2.998 ± 0.7267	3.237 ± 0.669	3.5 ± 0.67	0.0253^*∗*^
Platelets	181 ± 70.51	163 .6 ± 58.99	187.5 ± 82.29	186.4 ± 80.6	0.4622

## Data Availability

The datasets used and/or analyzed during the current study are available from the corresponding author upon reasonable request.

## Abbreviations

CKD: Chronic kidney disease
CRF: Chronic renal failure
CRP: C-reactive protein
CVD: Cardiovascular disease
EPO: Erythropoietin
ESRD: End-stage renal disease
GA: Gum Arabic
HD: Haemodialysis
MDA: Malondyaldeide
OS: Oxidative stress
ROS: Reactive oxygen species
RRt: Renal replacement therapy
TAC: Total antioxidant capacity
TNF*α*: Tumor necrosis factor *α*
TWBCs: Total white blood cells.
